# Deterioration of Sexual Health in Cancer Survivors Five Years after Diagnosis: Data from the French National Prospective VICAN Survey

**DOI:** 10.3390/cancers12113453

**Published:** 2020-11-20

**Authors:** Lorène Seguin, Rajae Touzani, Anne-Déborah Bouhnik, Ali Ben Charif, Patricia Marino, Marc-Karim Bendiane, Anthony Gonçalves, Gwenaelle Gravis, Julien Mancini

**Affiliations:** 1 INSERM, IRD, SESSTIM, Sciences Economiques & Sociales de la Santé & Traitement de l’Information Médicale, Equipe CANBIOS Labellisée Ligue Contre le Cancer, Aix Marseille Univ, 13009 Marseille, France; seguinl@ipc.unicancer.fr (L.S.); rajae.touzani@inserm.fr (R.T.); patricia.marino@inserm.fr (P.M.); marc-karim.bendiane@inserm.fr (M.-K.B.); 2 Department of Medical Oncology, Institut Paoli-Calmettes, Aix-Marseille Université, Inserm, CNRS, CRCM, 13009 Marseille, France; goncalvesa@ipc.unicancer.fr (A.G.); gravisg@ipc.unicancer.fr (G.G.); 3 Institut Paoli-Calmettes, SESSTIM U1252, 13009 Marseille, France; 4 VITAM—Centre de recherche en santé durable Quebec, Quebec, QC G1J0A4, Canada; ali.ben-charif.1@ulaval.ca; 5 APHM, INSERM, IRD, SESSTIM, Sciences Economiques & Sociales de la Santé & Traitement de l’Information Médicale, Equipe CANBIOS Labellisée Ligue Contre le Cancer, Hop Timone, BioSTIC, Biostatistique et Technologies de l’Information et de la Communication, Aix Marseille Univ, 13005 Marseille, France; julien.mancini@inserm.fr

**Keywords:** cancer survivors, sexual health deterioration, VICAN, sexuality

## Abstract

**Simple Summary:**

Cancer impacts sexual health (SH) even years after diagnosis, but long-term consequences are not fully documented, especially in cancers unrelated to sexuality. This study aimed to assess SH deterioration five years after diagnosis in a large population of cancer survivors. Our results show that 57.3% reported substantial SH deterioration. Substantial deterioration was reported in all cancer sites (from 27.7% in melanoma to 83.1% in prostate). Treatment type, cancer sequelae, and pain, as well as psychological consequences (depression and anxiety, especially for younger patients) were associated with substantial SH deterioration. Five years after diagnosis, the majority of cancer survivors reported SH deterioration. Interventions should be developed to improve sexual health regardless of cancer site. Particular attention should be paid to depression and anxiety, especially in younger survivors.

**Abstract:**

Little is known about cancer survivors’ sexual health (SH)—particularly, from well after diagnosis and in cancers unrelated to sexuality. This study aimed to assess SH deterioration five years after diagnosis. We analyzed data from the French national VIe après le CANcer (VICAN) survey. Six items from the Relationship and Sexuality Scale were used to assess SH. Respondents were grouped according to an ascending hierarchical classification in four clusters: strong, moderate, and weak deterioration or stable (WD, SD, MD, or St). Out of 2195 eligible participants, 57.3% reported substantial SH deterioration as either SD (30.8%) or MD (26.5%), while WD and St accounted for 31.2% and 11.5% of respondents, respectively. Substantial deterioration was reported in all cancer sites (from 27.7% in melanoma to 83.1% in prostate). Treatment type, cancer sequelae, and pain, as well as psychological consequences (depression and anxiety, especially for younger patients) were associated with substantial SH deterioration. The same factors were identified after restricting the analysis to survivors of cancers unrelated to sexuality. Five years after diagnosis, the majority of cancer survivors reported SH deterioration. Interventions should be developed to improve SH regardless of cancer site. Particular attention should be paid to depression and anxiety, especially in younger survivors.

## 1. Introduction

There are more than three million people currently living in France either with cancer or in remission [1]. Studies on cancer survivors have shown that many face psychological, physical, and social challenges [2], including issues related to sexual health (SH) [3]. The World Health Organization defines SH as “a state of physical, emotional, mental and social well-being related to sexuality, not merely the absence of disease dysfunction or infirmity” [4]. Indeed, sexuality and intimacy are significantly associated with health-related quality of life for all ages, even among the elderly [5]. This is why sexual disorders in cancer survivors may, if they are not seriously taken into account, such as other sequelae, become permanent and potentially affect quality of life for many years [6].

The majority of studies related to the SH of cancer survivors have mainly involved pelvic and breast tumors [3,7]. In men, research has focused primarily on prostate and testicular cancers, and their corresponding impact on erectile dysfunction [3,8,9], whereas research in women has focused primarily on breast and gynecological cancers [10,11,12]. However, SH has also been shown to be impacted in other cancer sites, such as in colon–rectum cancer, which is the third most frequent cancer in France and may result in specific treatment-related sequelae [13,14]. SH problems were also identified in lung cancer [15], non-Hodgkin lymphoma [16], and head and neck cancer [17].

A few studies focusing on general populations of cancer survivors have highlighted that all age groups, genders, and cancer sites suffer from SH problems [18,19,20,21]. In France, Ben Charif et al. published a sexual health problems analysis in cancer survivors two years after diagnosis [18].

However, previous research on cancer survivors’ SH mainly focused on individual cancer types, which leads to limitations regarding the generalizability of the findings and comparisons between differing survivor populations. Indeed, a variety of standardized questionnaires have been used to assess sexual function in oncology settings [22], most of them were specific to only one type of cancer or gender and focused on the physical aspect (erectile function, lubrication in women), ignoring the other SH domains (sexual desire, hugging and kissing, orgasm difficulties). If a cancer treatment causes specific physical SH side effects such as fatigue, incontinence, erectile dysfunction, dyspareunia, problems with arousal, lubrication, and pain [6,12], then psychological consequences, such as a poor body image, depression, and anxiety, should also be prioritized in the care of cancer survivors [23,24,25], and could be strongly related to SH when viewed globally rather than through specific symptoms.

The “Relationship and Sexuality Scale (RSS)”, validated by Berglund et al. [26] in premenopausal women with breast cancer, is useful when gathering a broader understanding of SH in cancer survivors of all ages and genders as it assesses not only sexual function (including sexual desire, etc.), but also sexual frequency (including satisfaction with the frequency of hugging and kissing, etc.).

Moreover, even though new sexual disorders can appear later on, especially after treatments such as radiotherapy that may cause delayed onset side effects [27], the majority of studies have had short-term follow-ups. Consequently, there are few data available on the SH of cancer survivors further from diagnosis. The five year threshold is a standard survival indicator, sometimes used to refer to cancer recovery [28], that is supposed to be associated with the return to normal life.

Our study aimed to address these limitations via a multifactorial global assessment of the associations between SH, psychological and physical cancer consequences, sociodemographic characteristics, and healthcare consumption in a large national representative sample of cancer survivors with a long-term follow-up (five years after diagnosis).

We also aimed to assess the deterioration of SH, to identify associated factors and to examine whether these factors affected all cancer survivors, including those of cancers unrelated to sexuality.

## 2. Materials and Methods

### 2.1. Study Design

The French VICAN survey (VIe après le CANcer) was conducted among cancer survivors two and five years after diagnosis. The participants in the VICAN survey included men and women living in metropolitan France, between the ages of 18 and 82 years upon diagnosis of their first malignant cancer in 2010–2011, with one of 12 specific cancer sites with good, intermediate, or poor prognosis. These sites account for 88% of cancer incidence in France with seven cancer sites potentially related to sexuality (breast, prostate, colon-rectum, bladder, kidney, cervical, and endometrial) and five cancer sites without any overt relation to sexual or reproductive function (thyroid, melanoma, upper aero-digestive tract (UADT), non-Hodgkin lymphoma and lung) [18]. One of the main objectives of the VICAN survey was initially to investigate the barriers to and drivers of patients’ return to work, we therefore overrepresented those aged <52 at diagnosis, as they were aged <54 at the time of the survey two years after diagnosis and therefore too young for retirement or early retirement schemes. Accordingly, we defined two age strata: 18–52 and 53–82 at diagnosis. This cut off of 52 years has been kept for the study of all other outcomes. The respondents were also beneficiaries of one of the three main French health insurance organizations, which cover more than 90% of the French population. Three types of data were collected utilizing the VICAN framework: (1) declarative data collected via questionnaires intended for the survivors participating in the study, which were disseminated two (2012) and five (2015–2016) years after the diagnosis; (2) baseline medical and clinical data collected via the healthcare teams; (3) the healthcare consumption data extracted from the SNIIRAM (Système National d’Information Interrégimes et l’Assurance Maladie or national health insurance information system) [29]. The study methodology and data collection procedure have been described elsewhere [30]. The VICAN study methodology was approved by three French national ethics commissions: the CCTIRS (Comité Consultatif sur le Traitement de l’Information en Matière de Recherche dans le Domaine de la Santé or Advisory Committee for Data Processing in Health Research, study No. 11–143), the ISP (Institut de Santé Publique or Institute of Public Health, study No. C11-63), and the CNIL (Commission Nationale de l’Informatique et des Libertés or French Commission on Individual Data Protection and Public Liberties, study No. 911290).

### 2.2. Data and Sample

Our population was entirely comprised of respondents to the second questionnaire, disseminated five years after their diagnosis. This questionnaire collected data on living conditions: sociodemographic characteristics, couples’ relationships, sexuality, medical comorbidities, quality of life, psychological outcomes (anxiety, depression), and perceived sequelae including fatigue and pain. We also included healthcare consumption data from the SNIIRAM.

### 2.3. Measurements

#### 2.3.1. Sexual Health Evaluation

Patient sexual health after cancer diagnosis was evaluated using six items from the Relationship and Sexuality Scale (RSS), validated by Berglund et al. [26]. Although this scale was initially developed and validated for pre-menopausal women with breast cancer, the items are not specific to this population and have already been used in assessing relationships and sexuality in both genders and all ages, with or without cancer [18,31].

Three of the six items from the RSS assess sexual function and measure the level of deterioration in sexual desire after the illness (3: increased, 2: unchanged, 1: decreased, and 0: nonexistent), the ability to have an orgasm and the frequency of sexual intercourse after the illness (4: greatly increased, 3: slightly increased, 2: unchanged, 1: slightly decreased, and 0: greatly decreased).

The other three items from the RSS assess frequency and measure patient satisfaction with the frequency of hugs and kisses and the satisfaction with the frequency of current intercourse (4: very much, 3: very, 2: enough, 1: little, and 0: not at all), and the frequency of intercourse during the previous two weeks (4: four times or more, 3: thrice, 2: twice, 1: once, and 0: not at all).

A “do not wish to answer” option was introduced in the five-year survey to take patient sensitivity into account. They were considered as missing data in the analysis.

An overall indicator of SH five years after cancer diagnosis was determined using a previous methodological approach [18]. Four different groups of participants identified using a cluster analysis were characterized by differing SH evolution according to their mean levels of RSS items. Such as in this previous study [18], the four clusters were named as follows: strong deterioration (SD), moderate deterioration (MD), weak deterioration (WD), and stable or no deterioration (St). SD, for example, reflects a general deterioration with a decrease of sexual desire, of the frequency of sexual intercourse and of the ability to reach orgasm, associated with a poor satisfaction with the frequency of hugs and kisses and sexual intercourse.

#### 2.3.2. Perceived Sequelae and Psychosocial Outcomes

Anxiety and depression were measured using the Hospital Anxiety and Depression Scale (HADS). This scale contains 14 items, 7 relating to the evaluation of depression and 7 relating to the assessment of anxiety. An anxiety/depression score of more than 10 identified participants suffering from symptoms of anxiety/depression [32].

Fatigue was measured using the subscale of the EORTC QLQ (European Organization for Research and Treatment of Cancer Quality of Life Questionnaire) [33]. On a 0–100 scale, a score of ≥ 40 indicated clinically significant fatigue.

Significant cancer sequelae were assessed via the following question “Generally speaking, do you keep any sequelae due to your illness?” (1 = yes, and they are very severe, 2 = yes, and they are severe, 3 = yes, but they are moderate, 4 = yes, but they are mild, and 5 = No, I don’t have any). We then merged the first two categories (yes, and they are very severe/yes, and they are severe) to create a two-level indicator (Yes vs. No) of significant sequelae.

Quality of life: The validated SF-12 questionnaire was used to assess mental (Mental Component Summary) and physical (Physical Component Summary) health [34]. Two scores (from 0 to 100) were calculated.

Pain was estimated using the following question: “During the past 15 days, have you felt pain?” The responses were grouped into three categories (often, sometimes, and never).

#### 2.3.3. Medical Data Collected via the SNIIRAM

Data regarding the treatments received (chemotherapy and radiotherapy) were collected from the health insurance databases. A distinction was made between initial treatments: chemotherapy and/or radiotherapy administered in the two years following diagnosis and later treatments (from two years to five years after diagnosis).

For breast and prostate cancers, hormone therapy was considered. A three-level variable was considered: “treated by hormone therapy”, “not treated by hormone therapy”, or “not concerned (sites other than prostate or breast)”.

Cancer progression was also used to estimate the pejorative evolution of cancer. It was defined as the occurrence of one of the following events: occurrence of metastasis (beyond 12 months after diagnosis), second cancer, death, admission to palliative care, or administration of treatments (chemotherapy, radiotherapy and/or targeted therapy) other than the initial treatment.

#### 2.3.4. Cancer Sites

Descriptive analyses were originally performed on the entire study population with all 12 cancer sites. Due to low numbers, sites were split into one of two categories: cancers potentially related to sexuality including prostate, colon-rectum, urological cancers (bladder and kidney), gynecological cancers (cervical and endometrial) and “others”. The latter category included all cancer sites without any link to sexual or reproductive function: thyroid, melanoma, UADT, non-Hodgkin lymphoma and lung. This categorization follows the same design as the analysis conducted at two years of diagnosis [18].

### 2.4. Statistical Analyses

First, a hierarchical agglomerative cluster analysis [18] enabled us to obtain four different clusters to characterize SH deterioration SD, MD, WD, and St. To increase the statistical power in multivariate analysis, we merged the latter clusters (WD and St) into a single group renamed “WDSt”, and we defined substantial deterioration as a merging of SD and MD.

Second, chi-squared and ANOVA tests were performed for categorical and continuous variables, respectively. To identify factors associated with the deterioration of SH, multinomial logistic models were used. Analyses were stratified by gender and age (younger defined as ≤52 years at diagnosis vs. older defined as >52 years at diagnosis), and systematic adjustments were made for cancer sites. A stepwise procedure was used to select statistically significant factors in a multivariate model (entry threshold, *p* < 0.20) and only the remaining associated factors with *p* < 0.05 were kept in the final model. The robustness of the results was tested via sensitivity analysis of the subsample of participants diagnosed with cancers unrelated to sexuality.

All analyses were weighted to ensure the representativeness of the data at a national level and were performed using the STATA software program, version 14.0 (StataCorp, College Station, TX, USA).

## 3. Results

### 3.1. Population Characteristics and Distribution of SH Deterioration

Of the 4174 patients who participated in the study 5 years after their diagnosis, 52.6% (N = 2195) responded to the six items of the RSS. Compared to respondents, the non-respondents were characterized by higher proportions of women, of participants older than 52 years old, and/or of participants who were single. They also presented higher proportions of survivors diagnosed with breast, lung, colon-rectum, UADT, and bladder cancers, and of sufferers of depression and clinically significant fatigue. No other differences were identified between respondents and non-respondents (Table 1).

Of the 2195 respondents, 59.6% (N = 1308) were women, 56.7% were 52 years old or younger at the time of diagnosis, and more than half of the study population had a breast or prostate cancer diagnosis (40.5% and 18.6%, respectively). During the survey, the majority of respondents were in a relationship (90.0%) and almost half of them had a professional activity (49.8%). Five years after diagnosis, 46.9% of participants suffered from clinically significant fatigue, 23.3% reported significant cancer sequelae, and 38.1% reported having frequent pain in the two weeks prior. Moreover, 12.6% and 45.8% of participants had significant symptoms of depression and anxiety respectively.

Based on the RSS items, over half (57.3%) of the participants had a substantial deterioration of their SH, either SD (30.8%) or MD (26.5%), whereas 31.2% and 11.5% of the participants presented WD or St, respectively.

Table 2 shows the distribution of RSS items. The median scores for the majority of RSS items were 1, except for the items concerning the satisfaction with frequency of intercourse and with frequency of hugging and kissing (median = 2). The median scores of the three groups were between 0 and 1 for participants whose SH had strongly deteriorated (SD) and were lower than among those who suffered from MD (median between 1 and 2) or WDSt (median between 2 and 3).

### 3.2. Deterioration of SH According to Age, Gender, and Cancer Site

**Figure 1:**
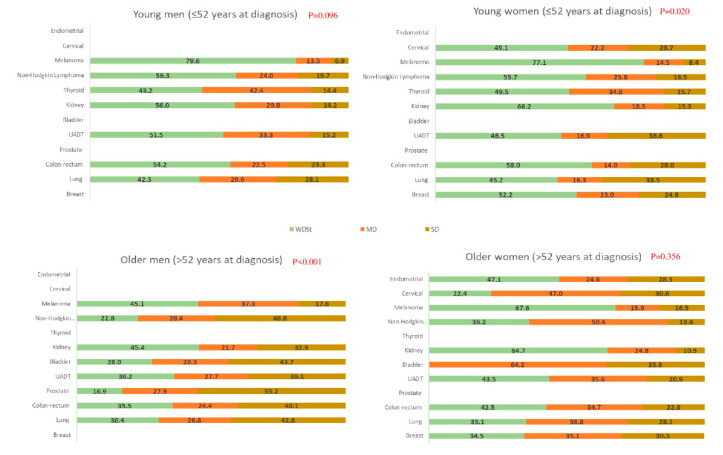
Deterioration of sexual health by age, gender and cancer site. WDSt: weak deterioration or stability, MD: moderate deterioration, SD: strong deterioration. For epidemiological reasons, prostate, bladder and endometrial cancers were only sampled in participants over 52 years old at diagnosis and thyroid cancers were only sampled in those less than 52 years.

Substantial deterioration (SD and MD) of patients’ SH was reported in all cancer sites (from 27.7% in melanoma to 83.1% in prostate). The deterioration of SH according to the cancer site was statistically significant in younger women (*p* = 0.020) and older men (*p* < 0.001) (Figure 1). Substantial deterioration of SH was observed for all cancer sites, regardless of gender and age. Rates ranged from 20.4% in younger men diagnosed with melanoma to 83.1% in older men diagnosed with prostate cancer (Figure 1). The proportion of SD was quite high in patients diagnosed with cancers related to sexuality; however, we also observed significant SD among survivors with cancers unrelated to sexuality. More specifically, substantial SH deterioration among older men was found in more than half of respondents; only melanoma, kidney, and UADT cancers had less than 40% SD. More than half of older women also suffered from substantial SH deterioration for all sites except melanoma and kidney, with more than 30% SD in cervical, breast, and bladder cancers. Among younger men, substantial deterioration was more often found in lung (57.7%) and thyroid (56.8%) cancers, with SD exceeding 20% in lung and colon-rectum cancers, while substantial deterioration among younger women was also found in both lung and thyroid cancers as well as cervical and UADT cancers (54.8%, 50.5%, 50.9% and 53.5% for lung, thyroid, cervical and UADT cancers respectively). SD was observed in more than 25% of younger women with cervical, UADT, colon-rectum and lung cancers. Moreover, within each group of participants, melanoma and kidney were the cancer sites most often associated with the lowest percentages of deterioration.

Based on these results, and those reported previously [18], the subsequent analyses were stratified according to age and gender (younger men N = 240, older men N = 647; younger women N = 1003 and older women N = 305).

### 3.3. Factors Associated with SH Deterioration According to Age and Gender

Univariate stratified analyses (Table 3 and Table 4) revealed that radiotherapy in both younger men and women, as well as chemotherapy in younger women, were associated with SH deterioration regardless of the period of administration. Chemotherapy administrated as an initial treatment was also associated with SH deterioration in older women but to a lesser extent. Hormone therapy was only relevant in older men. Unexpectedly, cancer progression since diagnosis was not significantly associated with SH deterioration (younger men *p* = 0.086; older men *p* = 0.413; younger women *p* = 0.099) except in older women (*p* < 0.001).

Depression, anxiety, fatigue, and significant cancer sequelae were all strongly associated with higher SH deterioration except in older women. For example, 30.7% of young male survivors who were anxious reported a SD compared to 14.2% in those without anxiety (in parallel 43.5% of anxious young male survivors reported WDst compared to 65.9% in those without anxiety). In addition, participants reporting SH deterioration were also associated with lower scores of both physical and mental health and higher frequencies of reported pain within the past 15 days, except in younger men.

The use of non-conventional medicine (NCM) was also associated with SH deterioration in women (younger: *p* = 0.041 and older: *p* = 0.048). Regarding co-morbidities, only arterial hypertension was significantly associated with SH deterioration among older men (*p* = 0.004) and younger women (*p* = 0.037).

The likelihood of SH deterioration increased among participants experiencing a decline in their couple’s relationship, including the elderly.

Independent factors associated with SD and MD are presented in Table 5, after multiple adjustment via multinomial regression models. Substantial levels of SH deterioration were significantly associated with the following factors: cancer site (prostate) in older men, age in younger women, depression in younger patients, anxiety in both older men and younger women, significant cancer sequelae in all patients except older women, use of NCM in younger women, pain in older women, and fatigue in older men.

Concerning the treatment types, the probability of SD increased in younger men who had received radiotherapy within the past three years and in older men who had received chemotherapy within the past three years. Chemotherapy was also associated with SD in younger women, while radiotherapy administered within the two years following diagnosis was associated with MD.

In addition to treatment type and consequences—such as significant cancer sequelae, fatigue, and/or pain—psychological consequences, such as depression and anxiety, were also important factors associated with substantial deterioration of SH in our sample, especially in younger patients. To ensure that the psychological consequences were not only related to the cancer site and specific treatment type (such as hormone therapy), we conducted a sensitivity analysis including only survivors diagnosed with cancers unrelated to sexuality, stratified by gender (Table S1 in supplementary data). The results remained consistent. In particular, no new factors were found to be associated with SD or MD in survivors of cancers unrelated to sexuality.

## 4. Discussion

### 4.1. Principal Findings

Our study assessed the sexual health of French cancer survivors in both non-sexuality and sexuality-related cancers, and demonstrated that the majority (57.3%) of survivors suffered from substantial SH deterioration even five years after diagnosis. The factors associated with SH deterioration were sociodemographic characteristics, such as age and gender (closely related to cancer type); significant cancer sequelae, such as fatigue and pain, treatment type; and psychological consequences, such as anxiety and depression.

### 4.2. The Majority of Participants Suffered from Substantial SH Deterioration, with No Improvement between Two and Five Years after Diagnosis

A previously published analysis [18] found that the majority of the 1955 sexually active French cancer survivors surveyed were affected by SH deterioration two years after their cancer diagnosis, and we observed the same result five years after diagnosis. There was even an increase in the proportion of SD at five years, as 30.0% of participants in both studies suffered from SD compared to 18.6% of participants two years after diagnosis (MD: 27.1% vs. 24.5% and WDSt: 42.9% vs. 56.9% at five years vs. two years respectively; *p* (paired Wilcoxon test) < 0.001). A previous comparison, among 1061 survivors who responded to the RSS items at both two and five years after diagnosis, revealed a decrease in sexual activity, the frequency of sexual intercourse, and the ability to reach orgasm [35]. While these results could reflect a response shift, i.e., a more pronounced perception of deterioration, they may also indicate that survivors are unable to cope with such long-term impairment of their SH. Further study is thus required to understand which survivors face such deterioration.

### 4.3. Medical Factors Associated with SH Deterioration Five Years after Diagnosis

Concerning cancer treatment, the likelihood of experiencing substantial SH deterioration increased in younger women who had received chemotherapy or radiotherapy, involving 53.5% and 69.1% of younger women in our sample, respectively.

Sexual disorders may appear later in patients who have undergone radiotherapy as a cancer treatment, especially in cervical, colorectal or breast cancers that are often treated with pelvic or breast radiotherapy [27]. A SD of SH was more often reported in younger men who had received radiotherapy within the past three years, and in older men who had received chemotherapy within the past three years (12.8% of older men in our sample). The use of radiotherapy at relapse in young men is quite rare (6% in our sample) and is mostly in cases of painful metastases [36], whereas chemotherapy at relapse in older men is essentially used for lung, non-Hodgkin lymphoma and colorectal cancers. Thus, the link between treatment at relapse and SH deterioration might be better explained by cancer severity regardless of the link to sexual or reproductive function.

In our study, SD was mostly observed in older men with prostate cancer, which is consistent with the high frequency of erectile dysfunction after prostate cancer treatments [37].

Since more than half of our sample was comprised of participants diagnosed with cancers related to sexuality (40.5% breast cancer and 18.6% prostate cancer) that are often treated with hormone therapy, the factors we identified as being associated with SH deterioration (significant cancer sequelae such as fatigue and pain, treatment type, and more importantly, psychological consequences, such as anxiety and depression) could all potentially be linked to hormone therapy, as suggested in other studies [38,39].

However, in our study, hormone therapy was significantly associated with deteriorating SH in only older men with prostate cancer according to bivariate analysis. Notably, among women with breast cancer (N = 409), only 16% were still receiving hormone therapy at the time of the interview, while 70% of men with prostate cancer (N = 889) were still receiving hormone therapy.

Although the deterioration of SH may be a direct consequence of cancer and its treatments, other comorbidities may have a major impact. In our study, there was an association between hypertension and SH deterioration, which has been previously described in the literature [40,41]. Diabetes has also been reported as an associated factor [42] but five-year data on diabetes was unavailable in our study sample.

Significant cancer sequelae were associated with SH deterioration in both genders, as was already observed two years after diagnosis [18]. Persistent sequelae as well as recurrent pain continued to disrupt patients’ sex lives even three years later. According to the literature, fatigue is known as one of the most common and distressing long-term consequences of cancer [43], and Götze et al. reported an association between fatigue and depression [24], which we then found to be itself linked to SH deterioration. In our study, fatigue was significantly associated with the SD of SH in older men, independently of reported sequelae and anxiety.

Non-conventional medicine (NCM) is known to be used by younger women, with higher levels of education, and both health problems and cancer diagnosis are often decisive factors in the use of NCM, as a pragmatic response to patient needs that conventional medicine failed to meet [44]. Indeed, we found that NCM usage was significantly associated with a SD of SH in younger women and in all women in the sample with cancers unrelated to sexuality. We can therefore hypothesize that the women who are more likely to use NCM resort to them because few “conventional” solutions are offered to them to improve their SH.

Ussher et al. [21], found that sex specific difficulties were the most commonly reported explanations for SH deterioration in women and men (vaginal dryness and erectile dysfunction, respectively). Interestingly, while we observed that women with cancers related to sexuality often suffered from vaginal dryness (33% and 38% with breast, endometrial and cervical cancers, respectively), we also found that patients with lung cancer often reported vaginal dryness (38%) as well. Similar observations were made concerning men and erectile dysfunction (61.1%, 38.3% and 36.2% in prostate and bladder cancer as well as lung cancer, respectively). (Figure S1). Sex specific difficulties should therefore be discussed with patients in order to offer both psychological and medicinal solutions.

### 4.4. Psychological Consequences, such as Anxiety and Depression, Have Major Impacts

Although sex specific difficulties largely explain the deterioration of SH, they are not the only explanation. Erectile dysfunction in particular is reportedly associated with psychological issues [45], and we ourselves found that 12.6% of participants were depressed (Table 1) which is close to the prevalence of depression that has been reported in cancer patients [24,46]. More specifically, Götze et al. [24] found that for two different patient cohorts (five vs. ten years after cancer diagnosis), women more often presented depression and anxiety than men (*p* < 0.001) with less depression and anxiety present in older patients (*p* < 0.001). Treatment type, including hormone therapy, was not associated with depression and anxiety.

In our study, depression and anxiety were strongly associated with SH deterioration in all patients, five years after diagnosis, and can be conceptualized as both causes and consequences of SH deterioration. The association between depression and SH was even more pronounced among the survivors of cancer unrelated to sexuality. As such, the evaluation of depression, which was not included in the previous two-year survey, is very relevant to understanding the importance of SH deterioration among survivors who sometimes suffer from specific sexuality-related issues but are often associated with a more general deterioration.

We decided to consider a sample of “cancers unrelated to sexuality”, in order to include patients whose cancers have no overt link to sex and reproduction but may still impact SH. Colon, bladder, and kidney cancers were not included in this group due to the potential for sequelae related to surgery or treatments that have an overt association with SH deterioration [13,18,47,48]. Moreover, this maintained the same design as the analysis conducted two years after the cancer diagnosis and allowed for a certain homogeneity in cancer site groupings (e.g., urology). Sensitivity analysis of the survivors of cancers unrelated to sexuality (lung, UADT, melanoma, thyroid and non-Hodgkin-lymphoma cancers) did not reveal any major differences in either regression models (Table S1 in supplementary data). Indeed, no additional factors were associated with SD or MD in this group.

While sociodemographic characteristics are also known to be associated with SH, such as a patient’s level of education [18,49], we focused instead on medical and psychosocial factors. However, it could be important to note that the majority of respondents to the RSS items (90%) were in a committed relationship, which is greater than in the general French population (66.4%) [50] and the deterioration of couples’ relationships was relevant to both younger and older patients as sexual activity and intimacy remain associated with quality of life, even in older adults [5].

### 4.5. Implications for the Care of Survivors and Future Perspectives

The majority of long-term French survivors (five years after cancer diagnosis) still suffered from SH deterioration in our study. However, in another study by Ben Charif et al., two years after cancer diagnosis, 54.7% of patients reported not having a single proposition to discuss SH, whereas patients with prostate or cervical cancer were more frequently able to discuss SH with their health care provider (56.3% and 39.6%, respectively) [51]. In the USA, the large majority of cancer centers reported having no sexual aids or other sexual health resources available for either men or women [52]. Thus, interventions should be developed to improve the SH of patients irrespective of age, gender, cancer site or severity of sexual problems. Furthermore, in addition to managing fatigue and general sequelae while encouraging therapeutic de-escalation whenever possible, oncosexology consultations and psychological support should also be systematically proposed, at least in major cancer centers. Careful attention must be paid to psychological consequences, especially since younger cancer survivors appear to be more affected by depression and anxiety, which are associated with SH deterioration.

E-health tools could be another solution, as they have already had important impacts in oncology regarding the access to information, diagnoses, and monitoring both during and post-treatment. As oncosexologists are rare, a web-based self-help program could be an interesting and modern option to reduce sexual dysfunction [53,54].

### 4.6. Strengths and Weaknesses

Our study had numerous strengths including a large sample size, national representativeness, and a relatively long-term follow-up. In addition, the sample size of survivors of cancers unrelated to sexuality was significant (N = 483) and analyses were based on detailed and reliable data from two different sources (patient-reported outcomes, health administrative databases). Unfortunately, the cross-sectional nature of the study constitutes a limitation, without any assessment of SH before the cancer diagnosis. The VICAN study itself also shares the general limitations of any approach using self-reported questionnaires, such as memory or social desirability bias. Specifically, on this subject, another limit concerns the high proportion of non-responses to the questions related to sexuality, particularly among women, older, and single survivors, due to the introduction of a “do not wish to answer” option in this new round of interviews. It may have underestimated the rates of SH deterioration [55]. Indeed, patients with sexual dysfunction may be embarrassed and reluctant to talk about it. In addition, single patients may suffer from an SH deterioration, but may not feel concerned by the topic as 90.0% of respondents to the RSS items were in a committed relationship vs. 49.9% of non-respondents. Although the RSS has been used in assessing relationships and sexuality in both genders and all ages, with or without cancer [31], it has not been validated for men and women with other types of cancer than breast cancer, and it constitutes a limitation.

There is a high level of variability across studies focusing on sexual dysfunction caused by methodologic differences in the instruments used to assess presence of sexual dysfunction, ages of participants, nature of samples, methodology used to gather the data, and cultural differences [56]. Comparison with the occurrence of sexual dysfunction in the background population would have been of interest but only one recent large study about sexual habits in France is available [57] and we could not compare our data as it particularly focused on sexual habits than sexual health. Moreover, the Relationship and Sexuality Scale evaluates the sexual health of a patient in comparison to that before the onset of cancer (or of another disease). Therefore, there is no comparison data available in general population for this scale.

## 5. Conclusions

We demonstrated that even five years after their cancer diagnosis, the majority of French cancer survivors reported sexual health deterioration. Interventions should be developed to improve the sexual health of patients regardless of their age, gender, or the site of their cancer. Particular attention should be paid to depression and anxiety, specifically in younger cancer survivors who appear to be more affected by these issues.

## Figures and Tables

**Table 1:** Characteristics of respondents and non-respondents to Relationship and Sexuality Scale (RSS) items, five years after diagnosis (N = 4174).

	Respondents N = 2195 (%)	Non-Respondents N = 1979 (%)	*p*
Gender			
Men	887 (40.4) 1308 (59.6)	685 (34.6)	<0.001
Women		1294 (65.4)	
Age at diagnosis			
Younger (18–52)	1245 (56.7) 950 (43.3)	772 (39.0)	<0.001
Older (53–82)		1207 (61.0)	
Living as a couple *			
Yes	1975 (90.0) 220 (10.0)	987 (49.9)	<0.001
No		992 (50.1)	
Cancer site			
Breast	889 (40.5)	825 (41.7)	<0.001
Lung	74 (3.4)	81 (4.1)	
Colon-rectum	193 (8.8)	228 (11.5)	
Prostate	409 (18.6)	283 (14.3)	
Upper aerodigestive tract	73 (3.3)	119 (6.0)	
Bladder	52 (2.4)	81 (4.1)	
Kidney	95 (4.3)	51 (2.6)	
Thyroid	140 (6.4)	75 (3.8)	
Non-Hodgkin lymphoma	81 (3.7)	79 (4.0)	
Melanoma	119 (5.4)	83 (4.2)	
Cervical	54 (2.5)	38 (1.9)	
Endometrial	16 (0.7)	36 (1.8)	
Anxiety *			
No anxiety	1190 (54.2)	1031 (52.1)	0.237
Anxiety	1005 (45.8)	948 (47.9)	
Depression *			
No depression	1919 (87.4)	1538 (77.7)	<0.001
Depression	276 (12.6)	441 (22.3)	
EORTC Fatigue (score ≥ 40) *			0.036
No	1166 (53.1)	976 (49.3)	
Yes	1028 (46.9)	1003 (50.7)	
Significant cancer sequelae *			0.719
No	1672 (76.7)	1530 (77.3)	
Yes	507 (23.3)	449 (22.7)	
Pain within the past 15 days *			
Often	835 (38.1)	762 (38.5)	
Sometimes	765 (34.9)	706 (35.7)	0.726
Never	593 (27.0)	511 (25.8)	

WDSt: weak deterioration or stable; MD: moderate deterioration; SD: strong deterioration; * at the time of the survey.

**Table 2:** Distribution of RSS items among respondents.

RSS Items *	Median [Range]	Wdst (42.7%)	MD (26.5%)	SD (30.8%)
Impact of disease on sexual arousal (0–3)	1 [0–3]	2 [0–3]	1 [0–3]	1 [0–3]
Decrease in frequency of intercourse (0–4)	1 [0–4]	2 [0–4]	1 [0–4]	0 [0–2]
Decrease in possibility to reach orgasm (0–4)	1 [0–4]	2 [0–4]	1 [0–4]	0 [0–4]
Satisfaction with frequency of intercourse (0–4)	2 [0–4]	3 [0–4]	2 [0–4]	0 [0–2]
Intercourse during previous two weeks (0–4)	1 [0–4]	3 [0–4]	1 [0–4]	0 [0–3]
Satisfaction with frequency of hugging and kissing (0–4)	2 [0–4]	3 [0–4]	2 [0–4]	1 [0–4]

WDSt: weak deterioration or stable; MD: moderate deterioration; SD: strong deterioration; * lower scores indicate poorer sexual health for all items.

**Table 3:** Univariate analysis of factors associated with SH deterioration in men five years after cancer diagnosis: (N = 887).

	Younger (N = 240)				Older (N = 647)			
	WDSt	MD	SD	*p*	WDSt	MD	SD	*p*
Age * *M(SD)*	43.7 (6.5)	43.9 (6.0)	44.6 (6.8)	0.320	63.7 (7.6)	65.1 (6.5)	65.6 (6.3)	<0.001
Psycho-Social Outcomes and General Sequelae Reported at Survey								
Anxiety				0.003				<0.001
No anxiety	60.9	28.1	11.0		27.2	29.3	43.5	
Anxiety	47.4	25.2	27.4		13.8	22.8	63.4	
Depression				<0.001				<0.001
No depression	62.0	25.3	12.7		25.6	29.1	45.3	
Depression	22.0	37.1	40.9		8.1	16.1	75.7	
EORTC Fatigue (score ≥ 40)				0.001				<0.001
No	62.2	28.6	9.2		28.3	31.2	40.5	
Yes	49.0	25.4	25.6		12.1	18.8	69.1	
Significant cancer sequelae				0.002				<0.001
No	61.1	25.2	13.7		29.8	28.8	41.4	
Yes	35.9	34.6	29.5		5.7	23.5	70.7	
Pain within the past 15 days				0.373				0.004
Often	56.2	21.3	22.5		14.6	29.2	56.2	
Sometimes	57.6	27.4	14.9		21.6	24.9	53.5	
Never	54.1	32.3	13.6		31.5	28.6	39.9	
Physical quality of life score *M(SD)*	47.7 (9.7)	47.8 (8.8)	44.2 (11.9)	0.213	50.2 (9.1)	48.5 (7.3)	44.3 (8.8)	<0.001
Mental quality of life score *(SD)*	48.6 (9.3)	45.4 (8.8)	39.7 (10.6)	<0.001	51.6 (7.5)	50.4 (7.5)	46.5 (9.8)	<0.001
Non-conventional medicine use				0.666				0.971
No	57.2	26.8	16.0		23.5	27.5	49.0	
Yes	51.1	27.5	21.4		22.1	27.8	50.1	
Since the diagnosis, the couple’s relationship has				<0.001				<0.001
Strengthened	64.5	19.0	16.5		29.5	23.5	47.0	
Stayed the same	53.8	35.0	11.2		24.0	30.0	46.0	
Deteriorated	15.8	34.5	49.7		1.6	16.5	81.9	
Medical Characteristics								
Cancer site				0.665				<0.001
Other ***	56.5	27.9	15.6		33.5	29.4	37.1	
Prostate	−	−	−		16.9	27.9	55.2	
Colon-rectum	54.2	22.6	23.2		35.5	24.4	40.1	
Urological ****	56.3	29.6	14.1		34.7	25.8	39.5	
Chemotherapy				0.066				0.134
Initial treatment	51.7	27.5	20.8		33.0	25.6	41.4	
In the past 3 years	48.3	23.5	28.2		17.1	23.8	59.2	
No	61.1	27.8	11.1		23.3	28.3	48.4	
Radiotherapy				0.003				0.326
Initial treatment	45.2	31.8	23.0		26.4	23.9	49.7	
In the past 3 years	37.6	17.8	44.6		11.7	30.2	58.1	
No	61.5	26.2	12.3		23.8	28.6	47.6	
Hormone therapy since diagnosis				NC				<0.001
Yes	NC	NC	NC		5.9	18.9	75.2	
No					18.8	30.0	51.2	
Not concerned					34.5	26.8	38.7	
Cancer progression since the diagnosis				0.086				0.413
No	58.9	27.0	14.1		24.7	27.7	47.6	
Yes	48.1	27.3	24.6		19.7	26.9	53.4	
Arterial hypertension **				0.754				0.004
No	55.8	27.8	16.4		24.8	32.2	43.0	
Yes	55.4	24.0	20.6		21.5	20.9	57.6	
Heart disorders **				0.603				0.154
No	56.4	27.3	16.3		23.5	29.4	47.1	
Yes	51.3	23.8	24.9		23.1	20.6	56.3	

Younger: age at diagnosis ≤ 52 years; Older: age at diagnosis > 52 years; WDSt: weak deterioration or stable; MD: moderate deterioration; SD: strong deterioration. All data are row percentages except when specified; M(SD): mean (standard deviation); NC: not concerned; * at the time of diagnosis; ** at the time of the survey; *** other: cancer without any link to sexual or reproductive function (lung, upper aerodigestive tract, melanoma, thyroid and non-Hodgkin lymphoma); **** urological: bladder/kidney.

**Table 4:** Univariate analysis of factors associated with sexual health (SH) deterioration in women five years after diagnosis: (N = 1308).

	Younger (N = 1003)				Older (N = 305)			
	WDSt	MD	SD	*p*	WDSt	MD	SD	*p*
Age * *(SD)*								
	41.8	42.6	44.8	<0.001	61.0	61.5	62.9	0.777
	(6.8)	(6.4)	(5.6)		(6.3)	(7.1)	(6.8)	
Psycho-Social Outcomes and General Sequelae Reported at Survey								
Anxiety				<0.001				0.687
No anxiety	65.9	19.9	14.2		40.7	34.8	24.5	
Anxiety	43.5	25.9	30.7		35.7	34.4	29.9	
Depression				<0.001				0.104
No depression	58.3	23.6	18.1		40.7	33.5	25.8	
Depression	17.2	20.2	62.6		18.4	41.4	40.2	
EORTC Fatigue (score ≥ 40)				<0.001				0.121
No	60.5	24.4	15.1		41.3	36.7	22.0	
Yes	48.6	22.4	29.0		32.5	31.8	35.7	
Significant cancer sequelae				<0.001				0.202
No	58.6	23.0	18.4		40.1	32.5	27.4	
Yes	37.8	24.3	37.9		23.6	46.2	30.2	
Pain within the past 15 days				<0.001				0.024
Often	42.6	23.2	34.3		31.9	29.3	38.8	
Sometimes	61.5	22.2	16.3		39.7	35.1	25.2	
Never	64.1	25.0	10.9		45.7	44.1	10.2	
Physical quality of life score *M(SD)*	47.7 (9.9)	45.9 (8.9)	41.5 (10.8)	<0.001	46.7 (9.1)	45.6 (10.0)	42.0 (8.8)	0.004
Mental quality of life score *M(SD)*	46.5 (9.5)	43.3 (9.6)	38.3 (11.3)	<0.001	48.1 (9.4)	46.3 (10.0)	44.8 (9.5)	<0.001
Non-conventional medicine use				0.041				0.048
No	56.3	22.9	20.8		37.6	39.0	23.4	
Yes	47.8	23.9	28.3		38.2	22.1	39.7	
Since the diagnosis, the couple’s relationship has				<0.001				0.016
Strengthened	63.1	20.7	16.2		38.7	33.0	28.3	
Stayed the same	53.8	26.9	19.3		41.5	36.5	22.0	
Deteriorated	6.1	21.3	72.6		4.9	44.0	51.1	
Medical Characteristics								
Cancer site				0.148				0.765
Other ***	56.6	26.5	16.9		48.7	32.3	19.0	
Breast	52.2	23.0	24.8		34.5	35.1	30.3	
Colon-rectum	58.0	14.0	28.0		42.5	34.7	22.8	
Urological ****	66.2	18.6	15.3		45.2	36.7	18.1	
Gynecological *****	49.1	22.2	28.7		40.6	30.5	28.9	
Chemotherapy				<0.001				0.011
Initial treatment	50.2	22.0	27.8		26.0	38.0	36.0	
In the past 3 years	42.4	22.0	35.6		62.9	12.5	24.6	
No	59.1	24.7	16.2		38.8	37.4	23.8	
Radiotherapy				0.002				0.933
Initial treatment	49.5	25.2	25.3		36.9	35.5	27.6	
In the past 3 years	44.9	18.4	36.7		49.8	21.5	28.7	
No	63.6	19.2	17.2		38.2	34.1	27.7	
Hormone therapy since diagnosis				0.172				0.406
Yes	51.1	21.7	27.2		31.4	38.8	29.8	
No	55.0	26.1	18.9		41.8	26.5	31.7	
Not concerned	56.2	23.6	20.2		44.3	33.4	22.3	
Cancer progression since the diagnosis				0.099				<0.001
No	53.7	24.2	22.1		31.2	39.6	29.2	
Yes	52.2	18.2	29.6		59.6	17.7	22.7	
Arterial hypertension **				0.037				0.948
No	54.8	22.9	22.3		37.4	35.4	27.2	
Yes	38.9	27.0	34.1		38.4	33.1	28.5	
Heart disorders **				0.916				0.199
No	53.5	23.1	23.4		38.6	35.6	25.8	
Yes	52.1	25.8	22.1		31.0	25.6	43.4	

Younger: age at diagnosis ≤ 52 years; Older: age at diagnosis > 52 years; WDSt: weak deterioration or stable; MD: moderate deterioration; SD: strong deterioration. All data are row percentages except when specified; M(SD): mean (standard deviation); * at the time of diagnosis; ** at the time of the survey; *** other: cancer without any link to sexual or reproductive function (lung, upper aerodigestive tract, melanoma, thyroid and non-Hodgkin lymphoma); **** urological: bladder/kidney; ***** gynecological: endometrial and cervical.

**Table 5:** Multivariate analyses by multinomial logistic models of factors associated with SH deterioration according to age and gender five years after diagnosis.

**Men**	**MD**		**SD**		**Women**	**MD**		**SD**	
**Younger (N = 239)**	**ORa**	**95% CI**	**ORa**	**95% CI**	**Younger (N = 997)**	**ORa**	**95% CI**	**ORa**	**95% CI**
Cancer site					Cancer site				
Other ***	1				Other ***	1			
Prostate	-	-	-	-	Breast	0.65	0.37–1.15	0.87	0.46–1.63
Colon-rectum	0.82	0.41–1.61	1.54	0.73–3.28		*p* = 0.137		*p* = 0.670	
	*p* = 0.554		*p* = 0.257		Colon-rectum	0.50	0.25–1.01	1.13	0.47–2.71
Urological ****	1.34	0.53–3.38	1.38	0.43–4.36		*p* = 0.053		*p* = 0.782	
	*p* = 0.534		*p* = 0.584		Urological ****	0.61	0.22–1.75	0.72	0.15–3.39
Depression **						*p* = 0.362		*p* = 0.677	
No depression	1				Gynecological *****	0.79	0.36–1.71	1.39	0.60–3.22
Depression	3.60	1.45–8.97	7.58	3.05–18.83		*p* = 0.547		*p* = 0.443	
	*p* = 0.006		*p <* 0.001		Age *				
Significant cancer sequelae **					*Continuous variable*	1.03	1.01–1.06	*1.11*	*1.07–1.15*
No	1					*p* = 0.033		*p <* 0.001	
Yes	2.18	1.01–4.74	2.87	1.32–6.25	Anxiety **				
	*p* = 0.048		*p* = 0.008		No anxiety	1			
Radiotherapy					Anxiety	1.90	1.28–2.84	2.50	1.64–3.81
Initial treatment	1.55	0.77–3.15	1.97	0.90–4.31		*p* = 0.002		*p <* 0.001	
	*p* = 0.221		*p* = 0.089		Depression **				
In the past 3 years	1.03	0.22–4.79	5.14	1.73–15.28	No depression	1			
	*p* = 0.971		*p* = 0.003		Depression	2.04	0.96–4.35	7.84	4.12–14.89
No	1					*p* = 0.064		*p <* 0.001	
**Men**	**MD**		**SD**		Significant cancer sequelae **				
**Older (N = 639)**	**ORa**	**95% CI**	**ORa**	**95% CI**	No	1			
Cancer site					Yes	1.40	0.86–2.27	1.99	1.25–3.17
Other ***	1					*p* = 0.174		*p* = 0.004	
Prostate	1.95	1.02–3.75	4.77	2.49–9.16	Chemotherapy				
	*p* = 0.044		*p <* 0.001		Initial treatment	1.02	0.65–1.58	1.92	1.19–3.09
Colon-rectum	0.81	0.35–1.83	1.13	0.52–2.42		*p* = 0.942		*p* = 0.007	
	*p* = 0.606		*p* = 0.761		In the past 3 years	1.09	0.64–2.56	2.33	1.10–4.91
Urological ****	0.88	0.43–1.81	1.25	0.62–2.53		*p* = 0.485		*p* = 0.026	
	*p* = 0.732		*p* = 0.539		No	1			
Anxiety **					Radiotherapy				
No anxiety	1				Initial treatment	1.89	1.14–3.12	1.74	0.99–3.06
Anxiety	1.51	0.79–2.90	2.40	1.32–4.37		*p* = 0.013		*p* = 0.056	
	*p* = 0.210		*p* = 0.004		In the past 3 years	1.09	0.37–3.22	1.53	0.53–4.37
EORTC Fatigue (score ≥ 40) **						*p* = 0.880		*p* = 0.430	
No	1				No	1			
Yes	1.37	0.71–2.65	3.49	1.96–6.19	Non-conventional and complementary medicine use **				
	*p* = 0.344		*p <* 0.001		No	1			
Significant cancer sequelae **					Yes	1.20	0.82–1.78	1.73	1.14–2.63
No	1					*p* = 0.345		*p* = 0.010	
Yes	3.37	1.40–8.07	5.63	2.51–12.62	**Woman**	**MD**		**SD**	
	*p* = 0.007		*p* < 0.001		**Older (N = 305)**	**ORa**	**95% CI**	**ORa**	**95% CI**
Chemotherapy					Cancer site				
Initial treatment	1.04	0.47–2.28	1.44	0.67–3.08	Other ***	1			
	*p* = 0.920		*p* = 0.344		Breast	1.54	0.71–3.33	2.07	0.84–5.11
In the past 3 years	1.52	0.66–3.52	2.52	1.11–5.71		*p* = 0.269		*p* = 0.115	
	*p* = 0.325		*p* = 0.027		Colon-rectum	1.22	0.44–3.37	1.65	0.48–5.69
No	1					*p* = 0.699		*p* = 0.423	
					Urological ****	1.24	0.34–4.52	0.85	0.16–4.37
						*p* = 0.744		*p* = 0.844	
					Gynecological *****	1.12	0.45–2.81	2.18	0.72–6.54
						*p* = 0.806		*p* = 0.165	
					Pain within the past 15 days **				
					Often	0.91	0.38–2.18	5.39	1.66–17.49
						*p* = 0.829		*p* = 0.005	
					Sometimes	0.91	0.39–2.09	2.83	0.86–9.31
						*p* = 0.816		*p* = 0.087	
					No	1			

Reference modality = WDSt.Younger: age at diagnosis ≤ 52 years; Older: age at diagnosis > 52 years; WDSt: weak deterioration or stable; MD: moderate deterioration; SD: strong deterioration; * at the time of diagnosis; ** at the time of the survey; *** other: cancer without any link to sexual or reproductive function (lung, upper aerodigestive tract, melanoma, thyroid and non-Hodgkin lymphoma); **** urological: bladder/kidney; ***** gynecological: endometrial and cervical.

## Supplementary Materials

The following are available online at https://www.mdpi.com/2072-6694/12/11/3453/s1, Figure S1: Vaginal dryness and erectile dysfunction according to cancer site—Erectile dysfunction and vaginal dryness were highly associated with SH deterioration, with focus to medical issues we decided not to include this variable in the regression model, Table S1: Multinomial logistic models of factors associated with SH deterioration in participants diagnosed with cancers without any overt link to sexual or reproductive function (lung, upper aero-digestive tract (UADT), melanoma, thyroid and non-Hodgkin-lymphoma) (N = 486).
