# Supplementary material for: Deterioration of Sexual Health in Cancer Survivors Five Years after Diagnosis: Data from the French National Prospective VICAN Survey
Source: Cancers (Basel). 2020 Nov 20;12(11):3453. doi: 10.3390/cancers12113453 (PMC7699784; doi:10.3390/cancers12113453)
Supplement: Supplementary file 1 [file cancers-12-03453-s001.pdf]

# Supplementary Materials:

# Deterioration of Sexual Health in Cancer Survivors Five Years after Diagnosis : Data from the French National Prospective VICAN Survey

**Lorène Seguin [1,2], Rajae Touzani [1,3], Anne-Déborah Bouhnik [1,*], Ali Ben Charif [4], Patricia Marino [1,3], Marc-Karim Bendiane [1], Anthony Gonçalves [2], Gwenaelle Gravis [2] and Julien Mancini [5]**

**Table S1.** Multinomial logistic models of factors associated with SH deterioration in participants diagnosed with cancers without any overt link to sexual or reproductive function (lung, upper aero-digestive tract (UADT), melanoma, thyroid and non-Hodgkin-lymphoma) (N = 486).

| | **Men (N = 249)** | | | |
|---|---|---|---|---|
| | **MD** | | **SD** | |
| | ORa | 95% CI | ORa | 95% CI |
| Age at diagnosis | | | | |
| Younger | | | 1 | |
| Older | **1.98** | **1.16–3.39** | 4.72 | 2.47–9.02 |
| Depression * | | | | |
| No depression | | | 1 | |
| Depression | **3.32** | **1.21–9.10** | **13.26** | **5.24–33.54** |
| Significant cancer sequelae * | | | | |
| No | | | 1 | |
| Yes | **3.35** | **1.54–7.28** | 4.65 | **2.14–10.10** |
| Chemotherapy | | | | |
| Initial treatment | 1.01 | 0.56–1.84 | 1.73 | 0.87–3.43 |
| In the past 3 years | 1.61 | 0.72–3.63 | **4.51** | **1.82–11.18** |
| No | | | 1 | |
| | Women (N = 234) | | | |
| | MD | | SD | |
| | ORa | 95% CI | ORa | 95% CI |
| Depression* | | | | |
| No depression | | | 1 | |
| Depression | 1.96 | 0.59–6.57 | **7.44** | **2.30–24.03** |
| EORTC Fatigue (score ≥ 40) * | | | | |
| No | | | 1 | |
| Yes | 1.79 | 0.85–3.75 | **3.91** | **1.27–12.05** |
| Pain in the past 15 days * | | | | |
| Often | 1.32 | 0.53–3.28 | **4.23** | **1.09–16.49** |
| Sometimes | 0.86 | 0.37–2.00 | 2.05 | 0.46–9.05 |
| Never | | | 1 | |
| Chemotherapy | | | | |
| Initial treatment | 1.15 | 0.46–2.89 | **4.72** | **1.76–12.66** |
| In the past 3 years | **3.52** | **1.39–8.93** | 2.68 | 0.72–9.89 |
| No | | | 1 | |
| Radiotherapy | | | | |
| Initial treatment | **3.34** | **1.62–6.91** | 2.09 | 0.82–5.36 |
| In the past 3 years | 1.02 | 0.20–5.32 | 1.16 | 0.20–6.63 |
| No | | | 1 | |

| | | | | | |
|---|---|---|---|---|---|
| Non-conventional medicine use * | | | | | |
| | No | | | 1 | |
| | Yes | **2.66** | **1.29–5.48** | **3.30** | **1.34–8.15** |

Reference modality: WDSt: weak deterioration or stable; MD: moderate deterioration; SD: strong deterioration; Younger: age at diagnosis ≤ 52 years; Older: age at diagnosis > 52 years; * at the time of the survey. Bold for significant associations.

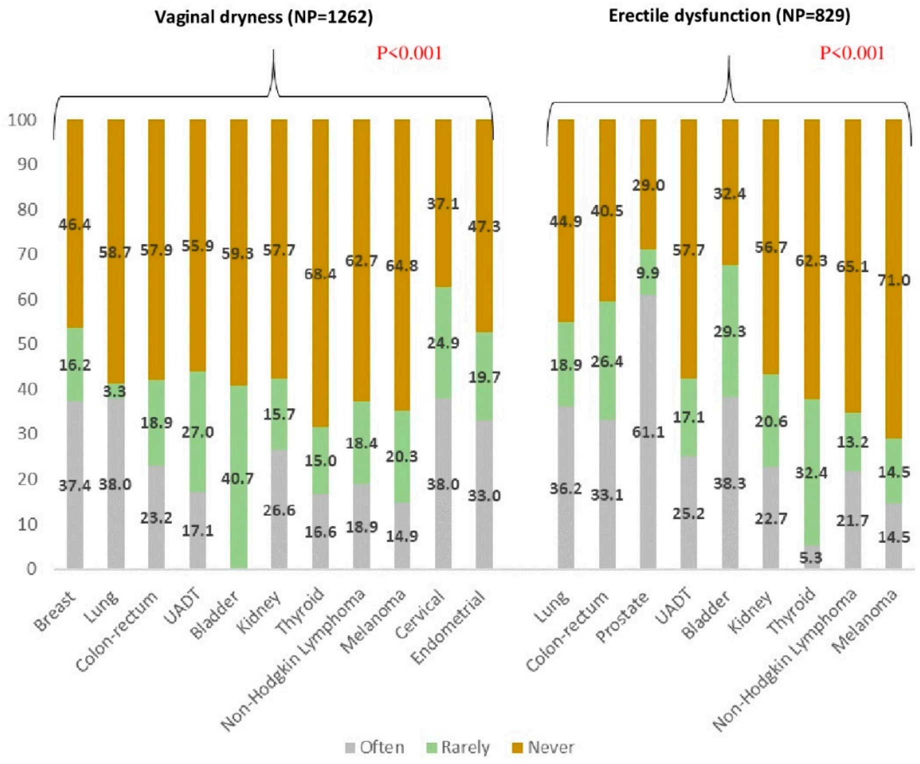


**Figure S1.** Vaginal dryness and erectile dysfunction according to cancer site. Erectile dysfunction and vaginal dryness were highly associated with SH deterioration, with focus to medical issues we decided not to include this variable in the regression model.
